# Detection and Characterization of Colistin Heteroresistance in an *Enterobacter cloacae* ST48 Strain Isolated From Raw Milk in Algeria

**DOI:** 10.1002/vms3.70501

**Published:** 2025-07-10

**Authors:** Sedrati Tahar, Touati Abdelaziz, Tennah Safia, Menoueri M. Nabil, Seydina M. Diene

**Affiliations:** ^1^ Laboratoire de recherche Gestion des Ressources Animales Locales Ecole Nationale Supérieure Vétérinaire Oued Smar Algérie; ^2^ Université Mohamed El Bachir El Ibrahimi de Bordj Bou Arreridj El Anceur Algérie; ^3^ Université de Bejaia Laboratoire d'Ecologie Microbienne FSNV Béjaïa Algérie; ^4^ Université SAAD DAHLAB de BLIDA route de Soumâa BP 270 Blida Blida Algérie; ^5^ MEPHI AP‐HM IHU‐Méditerranée Infection Faculté de Pharmacie Aix‐Marseille Univ. Marseille France

**Keywords:** colistin heteroresistance, *Enterobacter cloacae*, food products, mgrB, phoPQ, pmrAB

## Abstract

An *Enterobacter cloacae* ST48 isolate exhibiting a colistin‐heteroresistant phenotype was obtained from raw cow milk in Algeria. Two subpopulations were detected, both carrying the *bla*_TEM‐1_ gene. While one subpopulation (*E. cloacae* TS34‐a) was highly susceptible to colistin (MIC 0.06 µg/mL), the other (*E. cloacae* TS34‐b) was resistant (MIC 8 µg/mL). Population analysis profiling (PAP) confirmed heteroresistance. PCR amplification revealed the absence of *mcr* genes in both subpopulations. However, multiple non‐synonymous mutations, including a stop‐codon mutation in *phoP* gene, were found in the resistant subpopulation. This is the first report of colistin‐heteroresistant *E. cloacae* isolated from raw milk, underscoring the importance of vigilance in the dairy industry and food safety.

## Graphical abstract

*Enterobacter cloacae* ST48 isolate from raw cow milk exhibits colistin heteroresistnace profile.

Chromosomal gene mutations in mgrB, *phoPQ* and *pmrAB*, incude colistin resistance in *E. claocea* isolate

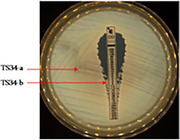

## 1 Introduction

*E. cloacae* is a Gram‐negative bacterium belonging to the family Enterobacteriaceae. It is an opportunistic pathogen capable of causing many infections in humans and animals, including urinary tract infections, respiratory tract infections, wound infections and bloodstream infections (Uechi et al. 2019). The clinical significance of *E. cloacae* has grown in recent years due to its ability to present substantial therapeutic challenges. These challenges arise from intrinsic and acquired resistance mechanisms to various antimicrobial agents, including Carbapenems (Hong et al. 2018). The global rise in infections caused by multidrug‐resistant (MDR) organisms, particularly carbapenemase‐producing Enterobacteriaceae (CPE), has significantly limited available treatment options (Hassani et al. 2022). Among the few viable alternatives, colistin, a member of the polypeptide family, has gained renewed clinical importance. Colistin exerts its antibacterial effects by targeting lipid A in the lipopolysaccharide (LPS) of the outer membrane of Gram‐negative bacteria (GNB), leading to bacterial cell death (Poirel et al. 2017). Although introduced in the 1950s, its clinical use waned due to concerns over nephrotoxicity and neurotoxicity. However, the alarming increase in carbapenem‐resistant infections has prompted a reassessment of colistin as a crucial last‐resort therapy for combatting MDR pathogens (Baron 2017). In recent years, the alarming emergence of resistance to colistin has been documented across various Gram‐negative bacterial species, including *E. cloacae*, *Klebsiella pneumoniae*, *Acinetobacter baumannii* and *Pseudomonas aeruginosa* (Loucif et al. 2022; Yousfi et al. 2019). The horizontal transfer of plasmid‐mediated colistin resistance genes has emerged as a significant concern. The *mcr* gene family, which currently includes *mcr*‐1 through *mcr*‐10, represents a transferable mechanism of colistin resistance detected globally across various bacterial species and environments (Luo et al. 2020). This trend poses a significant threat to the efficacy of colistin as a last‐resort antibiotic for MDR infections. Recent studies have further highlighted the rapid dissemination of these genes, particularly in clinical and agricultural settings, underscoring the urgent need for enhanced surveillance and alternative therapeutic strategies (Wang et al. 2022). In addition to plasmid‐mediated resistance, chromosomal mutations in genes such as *pmrA*, *pmrB*, *phoP* and *phoQ* have been identified as key contributors to colistin resistance. These mutations often lead to modifications in the bacterial outer membrane, reducing colistin binding and efficacy (Olaitan et al. 2016; Moffatt et al. 2019). Such resistance mechanisms are particularly concerning in clinical isolates, as they can arise spontaneously under colistin pressure, further complicating treatment options for MDR infections. Furthermore, heteroresistance, where a small subpopulation of bacteria within an otherwise susceptible population exhibits higher levels of antibiotic resistance, complicates detection and treatment strategies (Sánchez‐León et al. 2023). This phenomenon often goes undetected by standard antimicrobial susceptibility tests, leading to underestimation of resistance and potential treatment failures. Colistin heteroresistance in *E. cloacae* is driven by a complex interplay of genetic, phenotypic and metabolic mechanisms. Mutations in two‐component regulatory systems, such as *PhoP/Q* and *PmrA/B*, modify LPS, reducing colistin binding to the bacterial outer membrane. Notably, novel *PmrB* mutations have been identified that confer resistance without compromising bacterial fitness, highlighting the adaptability of these pathogens (Guérin et al. 2016). Overexpression of efflux pumps, particularly *AcrAB‐TolC*, facilitates the expulsion of colistin from bacterial cells and the use of efflux pump inhibitors has been shown to partially restore colistin susceptibility in heteroresistant isolates (Baron 2017). Metabolic adaptations, including the upregulation of the pentose phosphate pathway and increased production of reactive oxygen species scavengers, further bolster resistance. Additionally, small colony variants (SCVs) with altered cell wall composition and reduced membrane potential exhibit heightened antibiotic tolerance, contributing to the persistence of resistant subpopulations (Poirel et al. 2017). The activation of stress response mechanisms, such as the RpoS‐mediated general stress response, also plays a critical role in supporting the survival of heteroresistant subpopulations under colistin exposure (Charretier et al. 2018). Heteroresistance is further complicated by its association with persisters organisms capable of surviving antibiotic treatment without an increase in their minimal inhibitory concentration (MIC) and the selection of *stable mutants*, which exhibit a stable increase in MIC. Persisters represent an unstable subpopulation that can revert to susceptibility in the absence of antibiotic pressure, whereas stable mutants maintain elevated MIC levels due to genetic mutations, such as single‐nucleotide polymorphisms (SNPs), insertions or deletions. This dual dynamic of heteroresistance poses significant challenges for detection and treatment, as it can lead to persistent infections and the emergence of fully resistant strains under therapeutic pressure (Sánchez‐León et al. 2023). Recent studies have highlighted the growing prevalence of colistin heteroresistance in GNB from both clinical and environmental isolates (Jayol et al. 2015; Sánchez‐León et al. 2023). While heteroresistance has been extensively documented in pathogens such as *Klebsiella pneumoniae*, *Acinetobacter baumannii* and *Pseudomonas aeruginosa*, reports specifically addressing *Enterobacter cloacae* remain limited. Notably, to date, there have been no documented cases of colistin heteroresistance in *E. cloacae* isolated explicitly from food products, such as milk and dairy products. This gap in the literature underscores the need for further research to assess the potential role of foodborne transmission in the spread of colistin‐resistant *E. cloacae* and its implications for public health.

This study investigates the presence and characteristics of colistin heteroresistance in *E. cloacae* sequence type 48 (ST48), producing *bla*_TEM‐1_, isolated from raw milk in Algeria.

## 2 Materials and Methods

### 2.1 Bacterial Isolate and Antimicrobial Susceptibility Testing

An *E. cloacae* isolate was obtained from a raw milk sample collected from dairy cows in Algeria. 0.01 mL of milk was cultured on Columbia blood agar (COS, BioMerieux SA, France) and incubated for 24–48 h at 37°C. Pure colonies were selected and subcultured on Trypticase Soy Agar (TSA, BioMerieux SA, France) and bacterial isolate was identified by MALDI‐TOF mass spectrometry using the Microflex LT spectrometer and MALDI‐Biotyper 3.0 software (Bruker Daltonics, Bremen, Germany) as previously described (Singhal et al. 2015). Antimicrobial susceptibility testing was performed on 16 different antibiotics amoxicillin, amoxicillin‐clavulanic acid, piperacillin/tazobactam, cefalotin, ceftriaxone, cefepime, ertapenem, imipenem, fosfomycin, nitrofuran, trimethoprim/sulfamethoxazol, amikacin, ciprofloxacin, tetracycline, colistin and gentamicin (Bio‐Rad, Marnes‐la‐Coquette, France) using the agar disk diffusion method according to the guide‐lines of CASSFM/EUCAST 2019 (v.1.0) (http://www.sfm‐microbiologie.org/wpcontent/upload/2019/02/CASFM2019_V1.1.pdf). Additionally, colistin microdilution test (UMIC; Biocentric, Bandol, France) and E‐test (Biomerieux, France) were performed according to the manufacturer's instructions.

### 2.2 Detection of Heteroresistance

Colistin heteroresistance was investigated using the population analysis profile (PAP) method as described by (Andersson et al. 2019). Briefly, bacterial suspensions were prepared and plated on Mueller–Hinton agar containing various colistin concentrations (0, 0.5, 1, 2, 3, 4, 6 and 8 µg/mL). Colony counts were performed after 24 h of incubation at 37°C.

### 2.3 Molecular Characterization

The genomic DNA of the isolate was extracted with the EZ1 DNeasy Blood Tissue Kit (Qiagen GmbH, Kilden, Germany). Screening for genes encoding ESBLs (CTX‐M, TEM and SHV) and plasmid‐mediated colistin resistance genes (*mcr‐1* to *mcr‐8*) was carried out by real‐time polymerase chain reaction (RT‐PCR) with specific primers as previously described (Ngaiganam et al. 2019). Furthermore, investigation of chromosomal mutations in *mgrB* and those encoding for TCSs (*pmrA, pmrB, phoP* and *phoQ*) was performed by standard PCR using reported primers (Nawfal et al. 2020). The DNA from all samples that tested positive in PCR was cleaned and analysed using BigDye terminator sequencing technology on an ABI 3730xl machine (Applied Biosystems, Foster City, CA, USA). The genetic sequences obtained were then compared against the NCBI database using the BlastN program to identify matches. As previously described, multilocus sequence typing (MLST) was also conducted (Hong et al. 2018).

## 3 Results

### 3.1 Phenotypic Analyses

**FIGURE 1:**
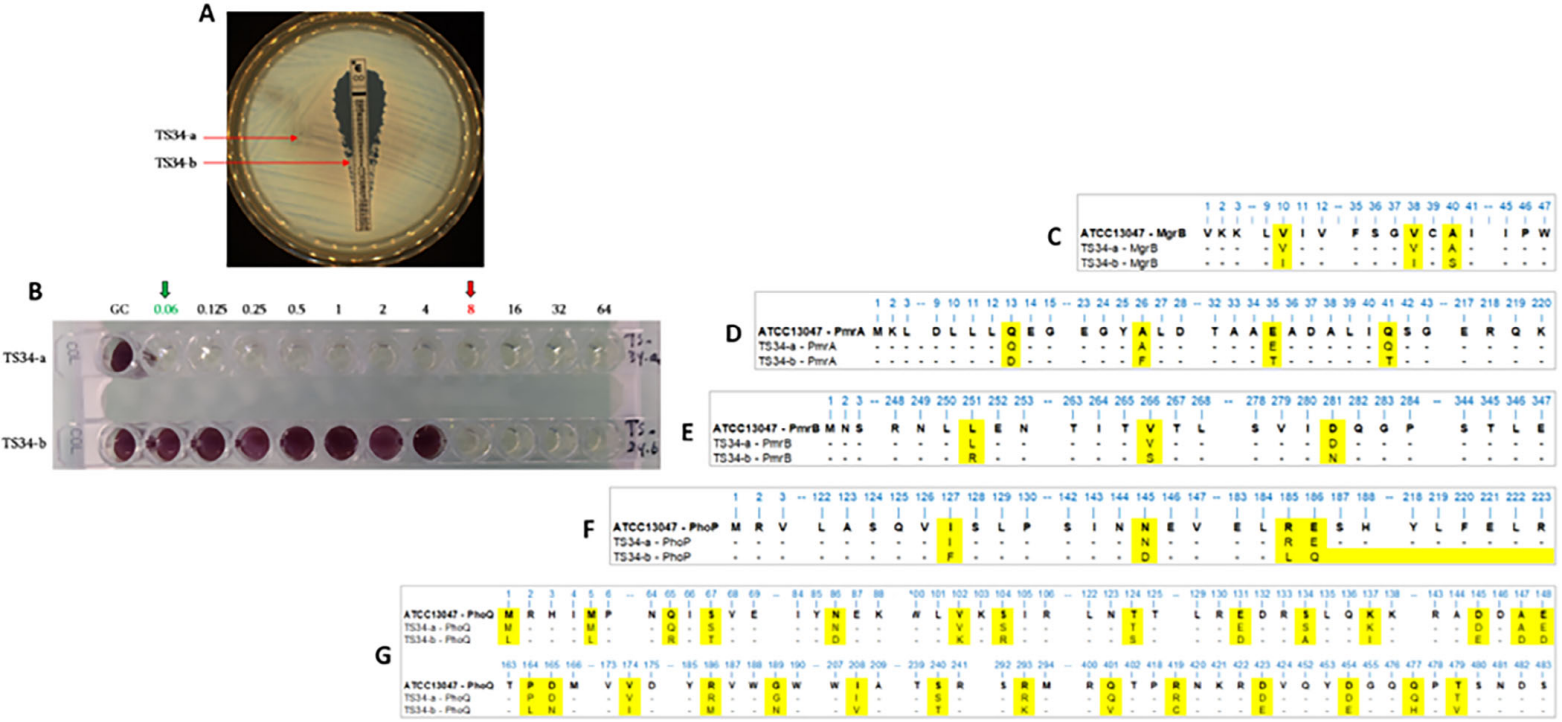
Phenotypic and molecular analysis of colistin resistance of studied *E. cloacae* isolate. (A) colistin susceptibility testing by E‐test of the heteroresistant *E. cloacae* isolate; (B) colistin susceptibility testing by microdilution of the separated two subpopulations (i.e., TS34‐a and TS34‐b), values refer to the tested colistin concentrations in µg/mL. Protein alignment colistin resistance proteins between subpopulations of colistin hetero‐resistant *E. cloacae* (TS34‐a and TS34‐b) with *E. cloacae* ATCC13047: (B) MgrB; (C) PmrA; (D) PmrB; (E) PhoP; and (F) PhoQ.

A pure colony was isolated from the bacterial culture and identified as *Enterobacter cloacae* using MALDI‐TOF MS, with an identification score ≥ 2.0. The antibiotic susceptibility testing of an isolated strain called *E. cloacae* TS34 reveals a resistance phenotype to amoxicillin, amoxicillin‐clavulanic acid, cefalotin, nitrofuran and colistin and a susceptible phenotype to the other tested antibiotics. Interestingly, the heteroresistance phenotype was observed around the colistin disk and this phenotype was confirmed by the performed colistin E‐test (Figure 1A). The two subpopulations called *E. cloacae* TS34‐a and TS34‐b were then subcultured several times on TSA medium and carefully separated and re‐identified as *E. cloacae* by MALDI‐TOF MS. Moreover, MLST analysis reveals that both subpopulations belong to the ST48 clone. By microdilution assay, colistin susceptibility of both strains was significantly different since the TS34‐a subpopulation was highly susceptible to colistin with an MIC of 0.06 µg/mL, whereas the TS34‐b subpopulation exhibits a colistin MIC of 8 µg/mL (Figure 1B; Table 1). Except for colistin, these two subpopulations exhibit the same antibiotic susceptibility profile (Table 1).

**TABLE 1:** Description of the two subpopulations of *E. cloacae* TS34‐a and TS34‐b isolates identified close to the E‐test strip.

Strain name	CT MIC (µg/mL)	Resistance phenotype	*bla* genes	Sequence type (ST)	Amino acid substitutions				
					MgrB	PmrA	PmrB	PhoP	PhoQ
***E. cloacae* TS34‐a**	0.06	AMX, AMC CF, F	*bla*_TEM‐1_	48	—	—	—	—	—
***E. cloacae* TS34‐b**	8	AMX, AMC CF, CT, F	*bla*_TEM‐1_	48	V10I,V38I, A40S	Q13D, A26F, E35T, Q41T	L251R, V266S, D281N	I127F, N145D, R185L, E186Q, Deletion of the rest of the protein	M1L, M5L, Q65R, S67T, N86D, V102K, S104R, T124S, E131D, S134A, K137I, D145E, A147D, E148D, P164L, D165N, V174I, R186M, G189N, I208V, S240T, R293K, Q401V, R419C, D423E, D454E, Q477H, T479V

Abbreviations: AMC, amoxicillin/clavulanic acid; AX, amoxicillin; CF, cefalotin; CT, colistin; F, nitrofuran.

### 3.2 Heteroresistance Profile

**FIGURE 2:**
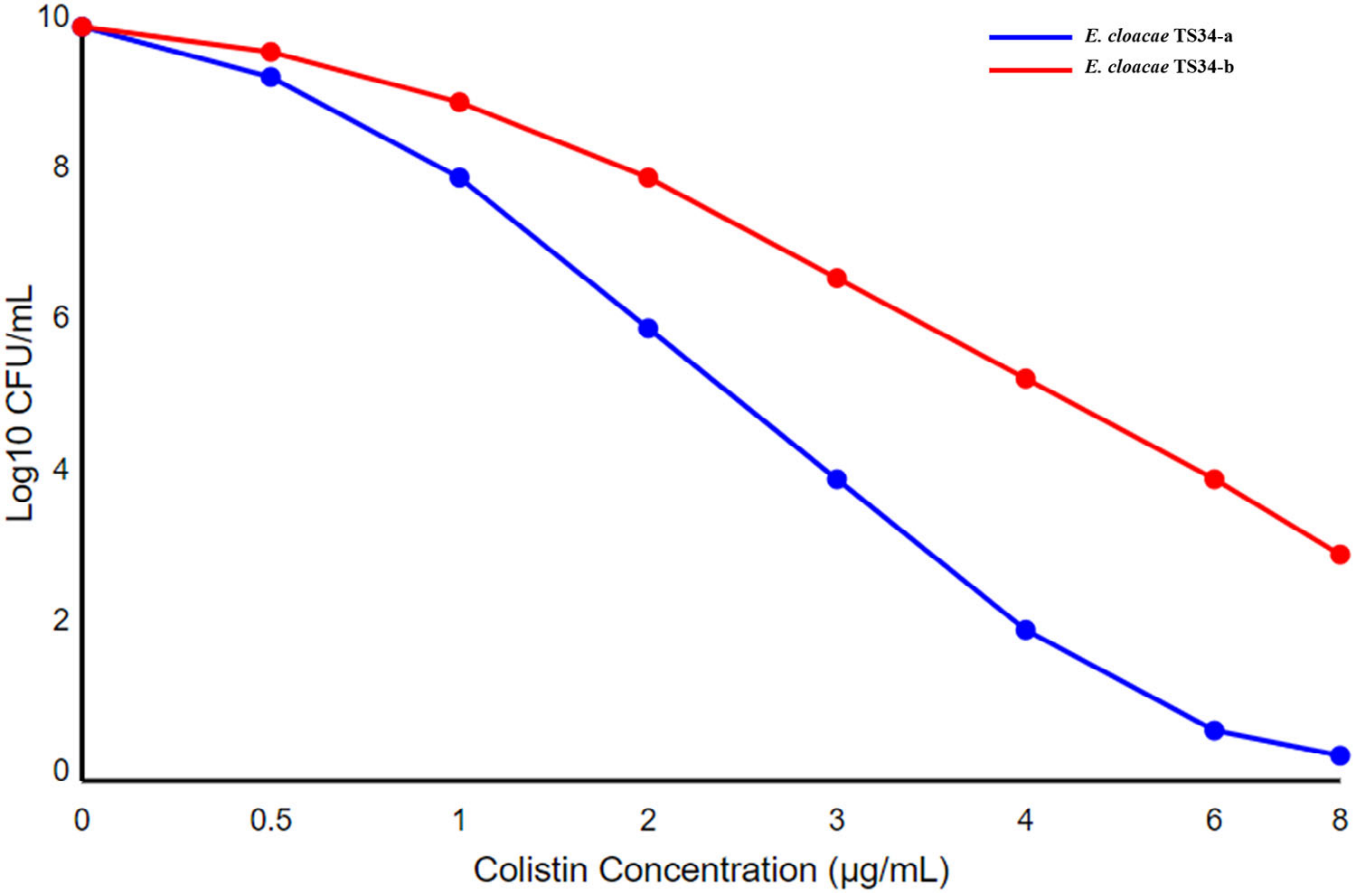
Population analysis profiles (PAPs) curves for the two subpopulations of *Enterobacter cloacae* isolates. The *x*‐axis represents the colistin concentration in µg/mL, ranging from 0 to 8 µg/mL. The *y*‐axis represents the bacterial count in Log10 CFU/mL, ranging from 0 to 8.

The PAP revealed that both subpopulations exhibited heteroresistance to colistin. Figure 2 shows the PAP curves for *E. cloacae* TS34‐a and TS34‐b, representing the log10 CFU/mL growth on different colistin concentrations relative to the plated viable counts. Both curves display a characteristic biphasic pattern indicative of heteroresistance, with a significant susceptible population and a minor resistant subpopulation. *E. cloacae* TS34‐b (red line) maintains higher viable counts at increased colistin concentrations than *E. cloacae* TS34‐a (blue line), consistent with its higher MIC value. The presence of colonies growing at colistin concentrations well above the MIC for both subpopulations further confirms the heteroresistance phenotype.

### 3.3 Molecular and Sequence Analyses

While RT‐PCR targeting *bla*_CTX‐M_ and *bla*_SHV_ was negative, *bla*_TEM_‐RT‐PCR was positive for both strains. The presence of *bla*_TEM‐1_ β‐lactamase was confirmed in both TS34‐a and TS34‐b isolates. No mobile colistin resistance genes, including *mcr*‐1 to *mcr*‐8, were detected in the TS34‐b isolate. Interestingly, while the sequenced genes from the colistin‐susceptible TS34‐a isolate were identical to those of the reference strain *E. cloacae* ATCC13047 (Figure 1C–F), the colistin‐resistant TS34‐b subpopulation exhibited several non‐synonymous mutations in the five investigated chromosomal‐associated colistin resistance genes (*mgrB*, *pmrA*, *pmrB*, *phoP* and *phoQ*) (Figure 1C–G). These mutations included a protein disruption in the PhoP protein (Figure 1F). All observed amino acid substitutions in the TS34‐b isolate are detailed in Table 1.

## 4 Discussion

*Enterobacter cloacae* is widely recognized as an environmental pathogen typically acquired from external sources such as soil, water, and plants. Its role as a causative agent of hospital‐acquired infections has been widely documented. However, the occurrence of *E. cloacae* in raw milk is less common. To date, no reports have documented colistin resistance or heteroresistance in *E. cloacae* isolated from raw milk. This could be because *E. cloacae* is typically not isolated from raw milk in significant numbers (Klaas and Zadoks 2018). Colistin heteroresistance often goes undetected with conventional antibiotic susceptibility testing (Poirel et al. 2017). The PAP results highlight a clear heteroresistance phenotype in *E. cloacae* TS34‐a and TS34‐b. The biphasic PAP curves reveal that while most bacterial populations are susceptible to colistin, a minor subpopulation can survive at higher colistin concentrations, characteristic of heteroresistance. Notably, *E. cloacae* TS34‐b shows more excellent resistance than TS34‐a, reflected in its higher MIC and ability to grow at elevated colistin levels. These findings underscore the limitations of traditional antibiotic susceptibility testing, which may fail to detect resistant subpopulations, leading to treatment failure. Our findings elucidate the molecular basis of colistin resistance and heteroresistance in the TS34‐b subpopulation of *E. cloacae*. These mechanisms are linked to specific amino acid alterations in genes encoding the two‐component regulatory systems, PmrAB and PhoPQ. Similar findings have been made in clinical settings. For instance, Uechi et al. (2019) found that single amino acid substitutions (I10V in MgrB), along with 15 substitutions in PmrA, 5 in PmrB, 6 in PhoP and 26 in PhoQ, contributed to colistin heteroresistance in *E. cloacae* isolates from patients in Japan (Uechi et al. 2019). Likewise, research conducted in Korea linked colistin resistance in *E. cloacae* isolates to multiple amino acid variations in PhoPQ and PmrAB proteins (Hong et al. 2018). Notably, heteroresistance mechanisms have also been described in other *Enterobacteriaceae* species. Jayol et al. (2015) found that a partial 25‐bp deletion in the *phoP* regulatory gene led to colistin heteroresistance in *K. pneumoniae*, indicating that gene alterations across different bacterial species may contribute to similar resistance phenotypes. Additionally, recent research provides further insights into colistin heteroresistance in *K. pneumoniae*, particularly in isolates producing OXA‐48. The study found substitutions in various proteins associated with colistin resistance, including PhoP, PhoQ, PmrB, PmrC, CrrA and CrrB, suggesting that colistin heteroresistance in *K. pneumoniae* involves multiple genetic pathways (Sánchez‐León et al. 2023). Our study found that 3332 subpopulations of *E. cloacae* (TS34‐a and TS34‐b) exhibited identical antibiotic susceptibility profiles, except for colistin. TS34‐a had an MIC of 0.06 µg/mL, while TS34‐b had a significantly higher MIC of 8 µg/mL. Both subpopulations shared identical protein profiles, as determined by MALDI‐TOF MS, and belonged to the same sequence type (ST48 clone). This suggests that the two subpopulations are isogenic but differ in their resistance to colistin, providing further evidence for the heteroresistance phenomenon. The PAP proved effective in detecting heteroresistance, which standard susceptibility testing methods might miss.

To our knowledge, this represents the first report of colistin‐heteroresistant *E. cloacae* isolated from raw cow's milk in Algeria. Furthermore, it is the first description of extensive genetic mutations in the *mgrB*, *pmrAB* and *phoPQ* genes involved in colistin resistance in *E. cloacae*. The detection of colistin resistance in raw milk of dairy cows is alarming and warrants immediate attention. Colistin has been widely used for decades in veterinary medicine for both prophylactic and therapeutic purposes, and its misuse has been associated with the emergence of colistin resistance mechanisms such as the *mcr‐1* gene and its homologs (Apostolakos and Piccirillo 2018). This research emphasizes the need for stricter surveillance and the careful use of colistin in animal farming to prevent the further spread of resistance. Given the importance of colistin as a last‐resort antibiotic in human medicine, monitoring resistance patterns in veterinary settings is crucial to prevent the crossover of resistance to human pathogens. Furthermore, the study highlights the importance of considering heteroresistance when evaluating antibiotic susceptibility, as standard testing methods may not detect resistant subpopulations. The findings in this study could contribute to policies aimed at reducing the misuse of antimicrobials and improving the surveillance of antibiotic resistance in livestock populations. However, this study has several limitations that should be acknowledged. It is based on a single heteroresistant *E. cloacae* isolate, which limits the generalizability of the results. In addition, while we identified non‐synonymous mutations in the *mgrB*, *pmrAB* and *phoPQ* genes, we did not conduct functional validation to confirm their direct role in colistin resistance. Future research involving larger sample sizes and functional assays is needed to validate these findings and provide deeper insights into the molecular mechanisms of heteroresistance.

## Author Contributions

**Sedrati Tahar**: conceptualization, methodology, formal analysis writing – original draft. **Touati Abdelaziz**: writing – review and editing. **Tennah Safia**: writing – review and editing. **Menoueri M. Nabil**: writing – review and editing. **Seydina M. Diene**: conceptualization, methodology, formal analysis, writing – review and editing.

## Ethics Statement

This conducted study here was approved by the research ethical comity of the university Mohamed El Bachir El Ibrahim de Bordj Bou Arreridj, under the number CER‐2025‐35.

## Conflicts of Interest

The authors declare no conflicts of interest.

## Funding

This work was supported by the Algerian Ministry of Higher Education and Scientific Research and by a grant from the French Government managed by the National Research Agency under the “Investissements d'avenir (Investments for the Future)” programme with the reference ANR‐10‐IAHU‐03 (Méditerranée Infection), by the Contrat Plan Etat‐Région and the European funding FEDER IHUPERF.

## Peer Review

The peer review history for this article is available at https://publons.com/publon/10.1002/vms3.70501.

## Data Availability

The data that support the findings of this study are available from the corresponding author upon reasonable request.
